# Influence of growth rates, microstructural properties and biochemical composition on the thermal stability of mycelia fungi

**DOI:** 10.1038/s41598-022-19458-0

**Published:** 2022-09-06

**Authors:** Nattanan Chulikavit, Tien Huynh, Chaitali Dekiwadia, Akbar Khatibi, Adrian Mouritz, Everson Kandare

**Affiliations:** 1 Aerospace Engineering and Aviation, School of Engineering, RMIT University, Bundoora, VIC 3083 Australia; 2 Biotechnology and Food Sciences, School of Science, RMIT University, Bundoora, VIC 3083 Australia; 3 RMIT Microscopy and Microanalysis Facility, RMIT University, Melbourne, VIC 3001 Australia

**Keywords:** Engineering, Materials science

## Abstract

Mycelium fungal species exhibit fire retardant characteristics. The influence of the growth media on the fungal growth rates, biochemical composition, and microstructural characteristics and their relationship to thermal properties is poorly understood. In this paper, we demonstrate that molasses can support the growth of non-pathogenic *Basidiomycota* phylum fungal species producing bio-derived materials with potential fire retardation characteristics. Scanning electron microscopy and Fourier transform infrared (FTIR) spectrometry were used to interrogate the microstructural and biochemical properties of the molasses-grown mycelia species. Thermal decomposition of molasses-fed mycelia was evaluated via thermogravimetric analysis interfaced with FTIR for real-time evolved gas analysis. The morphological and microstructural characteristics of the residual char post-thermal exposure were also evaluated. The material characterization enabled the establishment of a relationship between the microstructural, biochemical properties, and thermal properties of molasses-fed mycelia. This paper presents a comprehensive exploration of the mechanisms governing the thermal degradation of three mycelial species grown in molasses. These research findings advance the knowledge of critical parameters controlling fungal growth rates and yields as well as how the microstructural and biochemical properties influence the thermal response of mycelia.

## Introduction

The use of structurally efficient polymer composites in passenger-carrying vehicles and dwellings is constrained by stringent fire codes (e.g., material combustibility and flammability properties)^1^. Polymer composites ignite and burn with sustained flaming combustion when exposed to high temperatures and oxidative environments^2^. Burning polymer composites generate heat which can compromise the integrity of engineering structures through matrix softening, matrix decomposition, delamination cracking and fibre damage^3^. Further, burning polymers produce toxic gases and fumes such as carbon monoxide and partially decomposed hydrocarbons (i.e., carbon soot) which are responsible for most fire-related fatalities^4^. The 2017 Grenfell Tower fire, attributed to the use of polyethylene incorporated aluminium composite cladding panels that did not meet fire safety standards, resulted in 72 deaths—mostly caused by smoke inhalation^5^. Similarly, dense, toxic and irritant smoke from burning cabin materials caused 48 of the 55 deaths in the 1985 Manchester airport disaster in which the British Airtours Flight 28 M aircraft caught fire due to engine failure during take-off^6^. The Grenfell Tower fire and the Manchester airport disaster are just two examples of many fire tragedies highlighting the criticality of understanding fire reaction properties of polymers.

The integration of fire retardants (FRs) into the polymer composites effectively mitigates the flaming combustion reactions and reduces the volume of toxic gases and fumes^7,8^. There are several methods for integrating FRs into polymer composites including the modification of the polymer matrix using nano- and micro-sized FR particles^9^, the application of thermal protective surface coatings^10^, and the use of intrinsically fire retardant polymers such as phenolic resins^11^. For many years, halogenated compounds were FRs of choice for most polymer systems due to their highly efficient gas-phase fire retardation mechanisms^8,12^. Unfortunately, halogenated fire retardants release corrosive and ozone-depleting gases, limiting their use or resulting in removal in some jurisdictions^12,13^. The race to replace halogenated FRs has so far been dominated by both organic and inorganic phosphorus and nitrogen-containing compounds including ammonium polyphosphate^14^, melamine phosphate^15^, pentaerythritol^16^, intumescent compounds^17^, carbon-based nanomaterials (i.e. CNTs, graphene)^18^, metal salts^19^ and metal hydroxides^20^. While halogen-free FRs are effective, their widespread adoption is challenged by environmentally unfriendly manufacturing processes, occupational health and safety relating to the processing and handling of hazardous materials (i.e., carbon-based nanomaterials) and possible environmental damage due to heavy metal leaching. In contrast, bioderived FRs such as mycelium show potential for environmentally benign FRs that meet both the fire retardation and sustainable manufacturing requirements. However, the fire retardation efficacy of mycelium and corresponding fire retardation mechanisms are not yet fully understood to confidently inform large-scale application. When cultivating mycelium, it is critical that a sterile environment is maintained to prevent contamination by other pathogenic species. Maintaining a sterile growth environment at the industrial scale can be challenging. Further, product quality assurance will be challenged by batch variability due to the different growth patterns.

Mycelium is the vegetative part of fungi characterised by thread-like hyphae. It can be transformed from organic waste in an environmentally sustainable manner (e.g., produced at ambient conditions without the need for heat) to create biodegradable and naturally fire-resistant biomaterials. Due to the presence of chitin, protein and glucan in its cell walls, mycelium is inherently fire resistant and has excellent thermal stability. Chitin polymer chains contain *N*-acetyl glucosamine, a nitrogen source essential for generating NH_3_ gas which acts as a gas phase diluent that can suppress flaming combustion reactions^21^. Chitin and glucan have polysaccharide primary biochemical structural backbones, providing carbon which is essential for the generation of thermally protective surface char. The cysteine-rich protein components of mycelium (e.g., hydrophobins) contain disulphide bonds that break down at high temperatures to generate char-promoting hydrogen disulphide (H_2_S) molecules^22,23^. When used as a thermal protective film on flammable polymer composites, the residual char formed by the thermal degradation of mycelium acts as a thermal insulator protecting the underlying composite substrate.

Researchers have successfully used mycelium fungi to upcycle solid agricultural waste such as wheat grain^24^, rice husks^25^ and sawdust^26^ into fire-resistant biocomposite materials. However, some of the solid feed material remain undigested by mycelium. The partially digested residual solid feed particles can compromise the fire resistance and mechanical properties of the mycelium biocomposite. Limited studies have explored material properties of liquid media (e.g., molasses) fed mycelium fungi^27^. Unlike the solid feed particles that become inextricably integrated into the biocomposite, liquid feed material can be rinsed off. Further, after the retrieval of fully grown mycelium, the recovered liquid feed solution can be re-used to support the cultivation of fresh fungal albeit at slower growth rates due to depleted nutrients. Blackstrap molasses, a waste product from sugar refining, is a potential liquid feed material that has limited usage. Molasses has a high biomass and essential nutrients and can outperform commonly used malt extract in supporting mycelium growth^27^. Further, molasses contains calcium oxalate which decomposes into CaCO_3_ and CO at high temperatures^28^. CaCO_3_ further degrades into CaO and CO_2_. Both CO and CO_2_ are non-combustible gases that act as diluents snuffing out flaming combustion reactions during fire. Further, CaO enhances the thermal insulation efficacy of the residual surface char thereby thermally protecting the underlying polymer composite substrate. However, despite the numerous potential benefits of molasses as feed for mycelium, there is no research on its influence on the fungal growth rates, the biochemical composition and microstructure, and critically, the thermal and fire reaction properties of mycelium. Further, before mycelia can be integrated into engineering composites as a fire retardant or thermally protective layer, a comparative analysis against common polymer matrices is critical.

This project investigated the effects of molasses on the growth rates, mass yields and the thermal properties of three mycelia species selected from the non-pathogenic *Basidiomycota* phylum. Scanning electron microscopy (SEM) and Fourier transform infrared (FTIR) spectrometry were used for morphological, biochemical, and microstructural characterization of the pristine (denatured fungi) and post-thermal exposure residual char. The thermal decomposition of molasses-fed mycelia was evaluated via thermogravimetric analysis (TGA) interfaced with FTIR for real-time evolved gas analysis. The thermal stability of molasses grown mycelium was compared against wheat grain-fed fungi to assess the effect of feed type and residual undigested feed materials on thermal stability. Furthermore, the thermal stability of molasses fed mycelium was benchmarked against a commercial polymer matrix (epoxy) to assess its suitability as a matrix in biodegradable composites. Research findings from this work advance the understanding of critical parameters controlling fungal growth rates and mass yields and enable the establishment of an empirical correlation between the fungal microstructural, its biochemical properties and thermal stability.

## Materials and methods

### Fungal cultivation

Three fungal cultures *Ganoderma australe, Pleurotus ostreatus* and *Trametes versicolor* growing on malt extract agar were obtained from RMIT University’s Fungal Culture Collection (Australia) and were used as the inoculum. Molasses was purchased from E&A Salce (Australia). An epoxy resin (bisphenol A & F) (West System 105) and the corresponding hardener (West System 206) were supplied by Gougeon Brothers Incorporated (USA).

Molasses (15 g) was dissolved in water (135 g) making a 10 wt% molasses/water feed solution. To prevent contamination, the molasses/water solution was sterilised at 121 °C for 30 min and then cooled down to room temperature. A molasses/water solution (15 mL) was transferred at 20 °C to a sterile petri dish of 9 cm diameter. A 6 mm-diameter circular disc of the fungal culture was cut from the agar plate using the base of a sterile pipette. The inoculum was placed at the centre of the petri containing 15 mL of the molasses/water solution before sealing with Parafilm. At least three replicate specimens of each fungal species were cultivated. The sealed petri dish was incubated in a controlled environment (temperature 25 °C; 8-h white light cycle) for 10 days. Photographic images of the growing mycelium hyphal were taken at the same time on days 4, 7 and 10. The hyphal radial growth (cm^2^) was estimated using ImageJ software 1.46r (https://imagej.nih.gov/ij/).

### Mycelium and epoxy specimen preparation

The residual molasses and a brownish jelly-like film that formed during hyphal growth were washed off by rinsing the harvested mycelium film under running water for 30 min followed by soaking in warm water (50 °C) for 24 h. The mycelium film was repeatedly rinsed in water to remove residual sugar crystals from the molasses feed solution. The off-white mycelium film was deactivated by drying under vacuum bag pressure (− 1 atm) in an oven at 120 °C for 2 h. The dry mycelium film was stored in a hermetic plastic bag to prevent atmospheric moisture absorption. The epoxy resin and hardener were mixed according to the manufacturer’s recommended stoichiometric ratio and allowed to cure at room temperature for 24 h. The room temperature-cured epoxy matrix was post-cured at 60 °C for 8 h and used for benchmarking the thermal stability of mycelia.

### Chemical analysis

Mycelium films were characterised at room temperature using an Perkin Elmer (Spectra 100) Fourier Transform Infrared Spectrometer equipped with an attenuated total reflection (ATR) lens. The FTIR-ATR was used to identify functional groups in the pristine mycelia and in the solid residual char following the thermal degradation. For each FTIR-ATR experiment, 32 scans were acquired at 4 cm^−1^ resolution between 650 and 4000 cm^−1^ with the averaged spectrum recorded. The FTIR spectrum was baseline corrected and the absorption peaks were assigned using the FTIR-ATR in-built Perkin Elmer Spectrum 10.5.2 software. A minimum of three FTIR spectra were recorded for each fungi species.

### Thermogravimetric analysis (TGA) and evolved gas analysis (EGA)

The thermal-induced mass loss and the real-time FTIR analyses of evolved gases were carried out using TGA (Perkin Elmer STA 6000) interfaced with the Fourier Transform Infrared Spectrometer (Perkin Elmer Frontier). The mycelia films were conditioned at 60 °C for 3 h just before the TGA experiment to remove physically adsorbed moisture. The conditioning temperature of 60 °C was selected to preclude undesired thermal degradation of low molecular weight moieties. In a typical TGA experiment, micro-particles of mycelium or post-cured and crushed epoxy (~ 12 mg) were placed in an alumina crucible. The TGA furnace temperature was increased at a rate of 30 °C/min between 25 and 850 °C under flowing N_2_ gas (20 mL/min). The evolved gas transfer line connecting the TGA to the FTIR was maintained at 300 °C and was continuously flushed with N_2_ gas (flow rate of 50 mL/min) to prevent condensation of volatiles. The FTIR continuously analysed the evolved gases in the 4000 to 400 cm^−1^ wavenumber range with a 4 cm^−1^ resolution. The baseline curve obtained by conducting the TGA experiment with an empty sample holder was subtracted from all experimental datasets to compensate for buoyancy. The residual char yield was collected at 600 °C. A minimum of three experiments were conducted for each fungal species.

### Microstructural and morphological analysis

The microstructure and morphology of the mycelium (before and post-thermal degradation) were characterised using Scanning Electron Microscopy (FEI Qanta 200) equipped with an Energy Dispersive X-ray Spectrometer (SEM–EDS) operated by the Aztec software. SEM images were collected at an accelerating voltage of 15 kV, a spot size of 5 and a magnification of 3000. Pristine mycelium and TGA residual char samples (collected at 600 °C) were sputter coated with iridium using the Leica ACE600 sputter coater operated at 8 × 10^−3^ mbar achieving a coating thickness of approximately 5 nm. The hyphal filament radial diameter was estimated by analysing SEM images using ImageJ software 1.46r.

## Results and discussion

**Figure 1:**
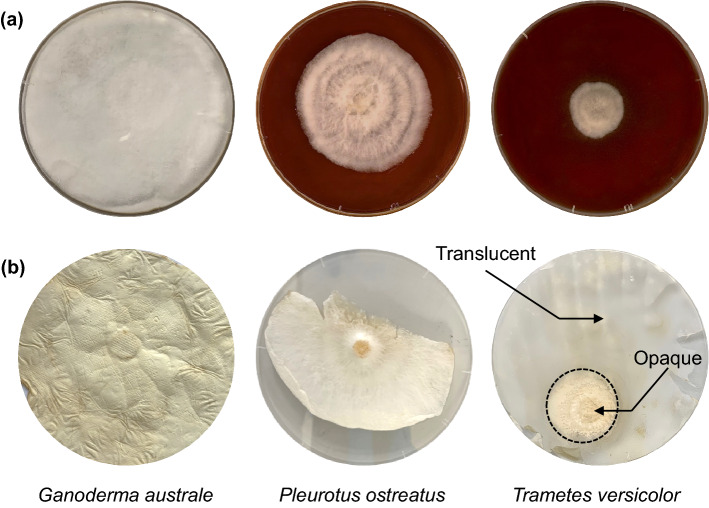
Growth and morphology of the *Ganoderma australe*, *Pleurotus ostreatus* and *Trametes versicolor* (**a**) live fungi on day 10 and (**b**) thermally deactivated mycelia films.

**Figure 2:**
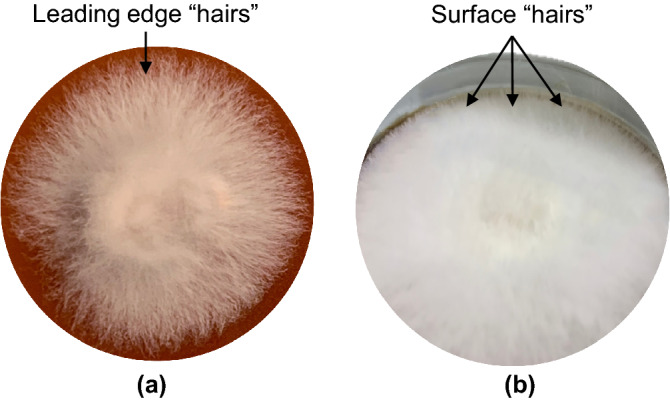
Morphology of hyphae revealing hair-like strands at the (**a**) leading edge and (**b**) air-exposed surface of *Pleurotus ostreatus*.

Photographic images of the live and denatured mycelia species revealed distinct surface topography as shown in Fig. 1. *Ganoderma australe* was characterised by a flat and fluffy topography while *Pleurotus ostreatus* and *Trametes versicolor* had undulating surfaces defined by radial ridges and valleys (Fig. 1a). Tiny hair-like strands, possibly surface-active and cysteine-rich proteins (hydrophobins) typically produced by filamentous fungi, were observed on mycelia films (Fig. 2). The hydrophobins layer enables the hyphae to breach the air-medium interface by reducing surface tension or preventing waterlogging while retaining permeability enabling gaseous exchange^22,23^. The image of *Ganoderma australe* taken on day 10 covered the entire surface area of the petri dish as shown in Fig. 1a. In contrast, the opaque hyphal section of the relatively slow-growing *Pleurotus ostreatus* and *Trametes versicolor* species respectively covered 43% and 7% of the petri dish surface.

Mycelium films were thermally deactivated via exposure to elevated temperature as described in the experimental section. The images of the denatured mycelia films are shown in Fig. 1b. The surface texture of the thermally deactivated films varied between the fungal species. *Ganoderma australe* was largely opaque and characterised by a rough surface texture. In contrast, the *Pleurotus ostreatus* and *Trametes versicolor* films revealed two distinct concentric regions; an opaque and rough section in the vicinity of the inoculation site at the geometric centre of the film as well as a larger, translucent, and relatively smoother peripheral section as indicated in Fig. 1b for *Trametes versicolor*. Like *Trametes versicolor, Pleurotus ostreatus* had a large translucent section. The dry mass yields of the thermally deactivated were highest for *Ganoderma australe,* followed by *Pleurotus ostreatus* and then *Trametes versicolor* weighing 168 ± 2, 77 ± 2 and 32 ± 3 mg, respectively. The normalised mass yields of *Pleurotus ostreatus* and *Trametes versicolor* relative to that of *Ganoderma australe* were 46% and 19%, respectively. It should be noted that the contribution of the translucent sections to the overall dry fungi mass yield was negligible.

**Figure 3:**
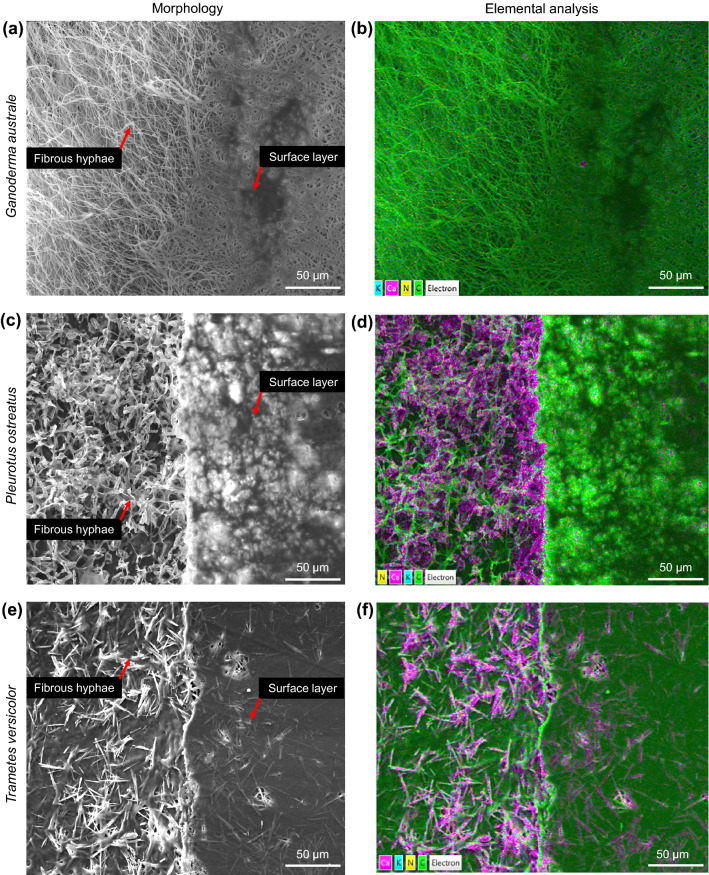
Morphology (left) and elemental analysis (right) of pristine mycelia—*Ganoderma australe* (**a**, **b**), *Pleurotus ostreatus* (**c**, **d**) and *Trametes versicolor* (**e**, **f**). The purple and green colours respectively represent calcium and carbon.

**Figure 4:**
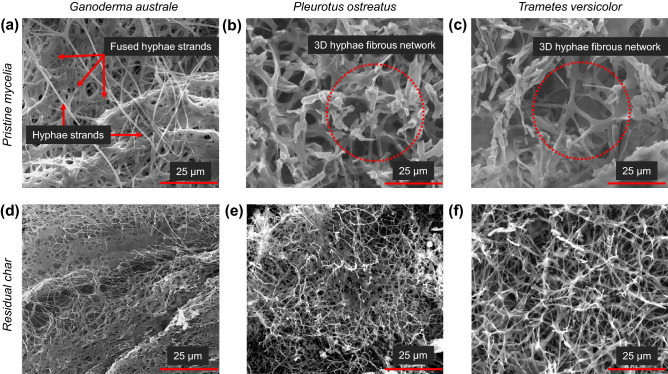
Hyphal morphology of pristine mycelia (top) and residual char (bottom) for *Ganoderma australe* (**a**, **d**), *Pleurotus ostreatus* (**b**, **e**) and *Trametes versicolor* (**c**, **f**).

All three fungal species revealed a porous (open cell-like foam) tubular hyphae filamentous network covered by continuous surface layers as shown in Fig. 3. The smooth surface layer texture may have resulted from consolidation of the hydrophobins layer during the thermal deactivation process. Elemental analysis of the three mycelia species revealed elemental variations between the fibrous hyphae network and surface layer, with a higher calcium concentration in the former. Molasses typically contains substantial amounts of calcium in the form of calcium oxalate. During fungal growth, calcium is incorporated into mycelia. The conversion of calcium oxalate into CaCO_3_, CO_2_ and CO has the potential to improve the thermal stability of mycelia^28^. The microstructure of the fibrous hyphae network varied between mycelia species as shown in Fig. 4. The hyphae strands in *Ganoderma australe* (Fig. 4a) were fused together, thinner but densely packed compared to those in *Pleurotus ostreatus* (Fig. 4b) and *Trametes versicolor* (Fig. 4c). Jones et al.^24^ reported that the fusion of *Trametes versicolor* hyphae fibrils required approximately 18 days of growth. In this study, *Trametes versicolor* and *Pleurotus ostreatus* were harvested on day 10, before hyphae fibril fusion. *Ganoderma australe* had fine fibrous hyphae that grew in a specific direction. SEM characterisation revealed noticeable microstructural differences between the opaque and translucent sections in *Pleurotus ostreatus* and *Trametes versicolor*. *Pleurotus ostreatus* had dense thickened hyphae with a random growth pattern while *Trametes versicolor* had crystalline shards and randomised growth.

**Figure 5:**
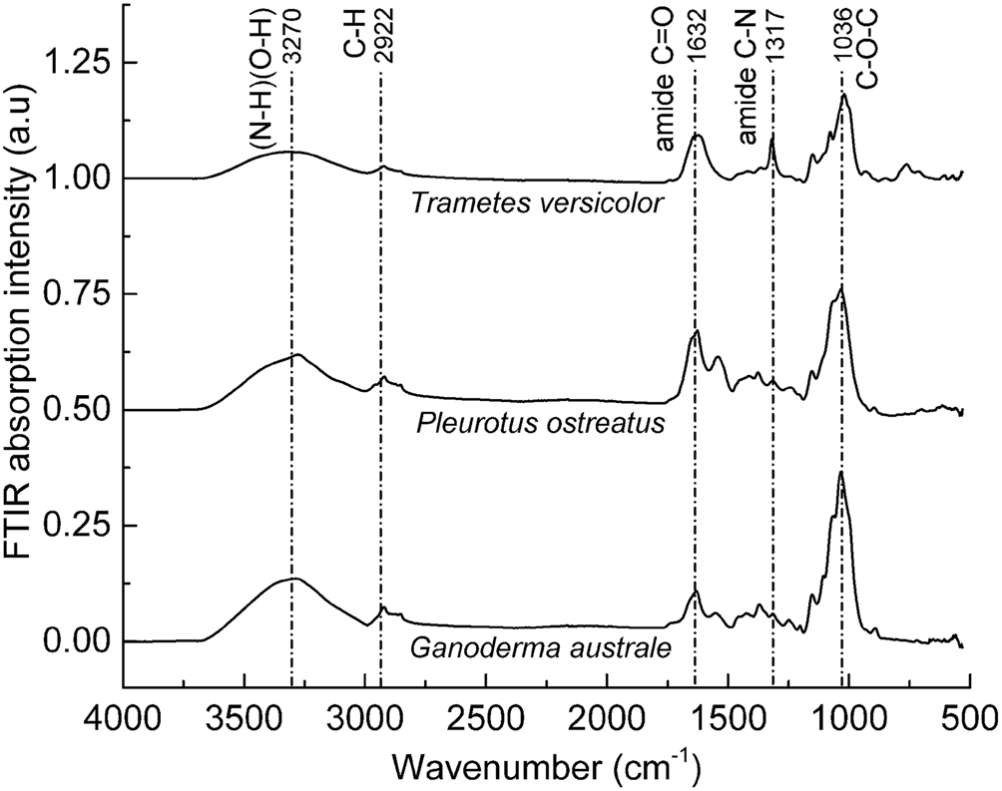
FTIR spectra of pristine mycelia species *Ganoderma australe*, *Pleurotus ostreatus* and *Trametes versicolor*. FTIR spectra were offset along the Y-axes but not scaled.

The thermally deactivated mycelia films were analysed using FTIR to establish the relationship between the hyphae microstructural features and the respective biochemical structure. There was little variation in the FTIR spectral analysis of the opaque sections of the three fungal species (Fig. 5). Since all three species are derived from the same *Basidiomycota* phylum, they are composed of chitin, protein and carbohydrates in the form of glucan and polysaccharides^29^. The species exhibited a broad absorption band centred at approximately 3330 cm^−1^ corresponding to the O–H stretching vibration in polysaccharides and/or the N–H stretching vibration of the amide functional group in proteins^30^. The broad and relatively weak absorption bands centred at approximately 2850 cm^−1^ and 2900 cm^−1^ were attributed to C–H stretching in chitin, protein and carbohydrates. The absorption peak around 1630 cm^−1^ was assigned to C=O stretching (amide I or amino acids)^31–33^, C=C stretching (amino acids)^31,34^ and/or N–H bending (flavonoids)^34^. The absorption band at approximately 1545 cm^−1^, which was prominent in *Ganoderma australe* and *Pleurotus ostreatus* was assigned to N–H bending (amide II) or C–N stretching (amide II)^30,35^. Relatively weak absorption bands were recorded for the three species at approximately 1317 cm^−1^ (amide III C–N stretching^32^; phenolic O–H stretching^33^), 1150 cm^−1^ (C–O stretching)^30,35,36^ and 1030 cm^−1^ (alcohol R–CH_2_–OH bending; C–O bending)^36^. A weak IR active band at around 760 cm^−1^ for the *Trametes versicolor* species was attributed to skeletal vibrations associated with the polysaccharide anomeric structure in glucan^37^.

Overall, the FTIR spectra of the three fungal species were characterised by five distinct absorption regions which are the N–H and O–H stretching (3700–3000 cm^−1^), C–H stretching (3000–2800 cm^−1^), protein amides (1750–1500 cm^−1^), polysaccharide (sugar) (1200–950 cm^−1^) and anomeric carbon (900–750 cm^−1^). FTIR spectral analysis confirmed the presence of chitin, protein and carbohydrates (i.e., glucan and polysaccharides), all of which possess inherent fire retardation characteristics^21,38,39^. The absorption peaks at 1750–1500 cm^−1^ (protein) and 1200–950 cm^−1^ (carbohydrate) can be used to compute the protein/carbohydrate ratio in mycelia. The protein/carbohydrate ratio can strongly influence the thermal degradation of mycelium, considering that carbon and nitrogen have distinct fire retardation mechanisms. The *N*-acetyl-*d*-glucosamine molecular chains in chitin generate diluent gases such as NH_3_ which suppress the flaming combustion reactions^40,41^. Conversely, carbon-rich polysaccharides (i.e., chitin and glucan) promote the formation of a thermally protective carbonaceous char^42^. The intra- and inter-molecular cysteine disulphide bonds in the hydrophobins layer can breakdown at high temperatures generating char-promoting hydrogen disulphide (H_2_S) molecules^22^.

**Figure 6:**
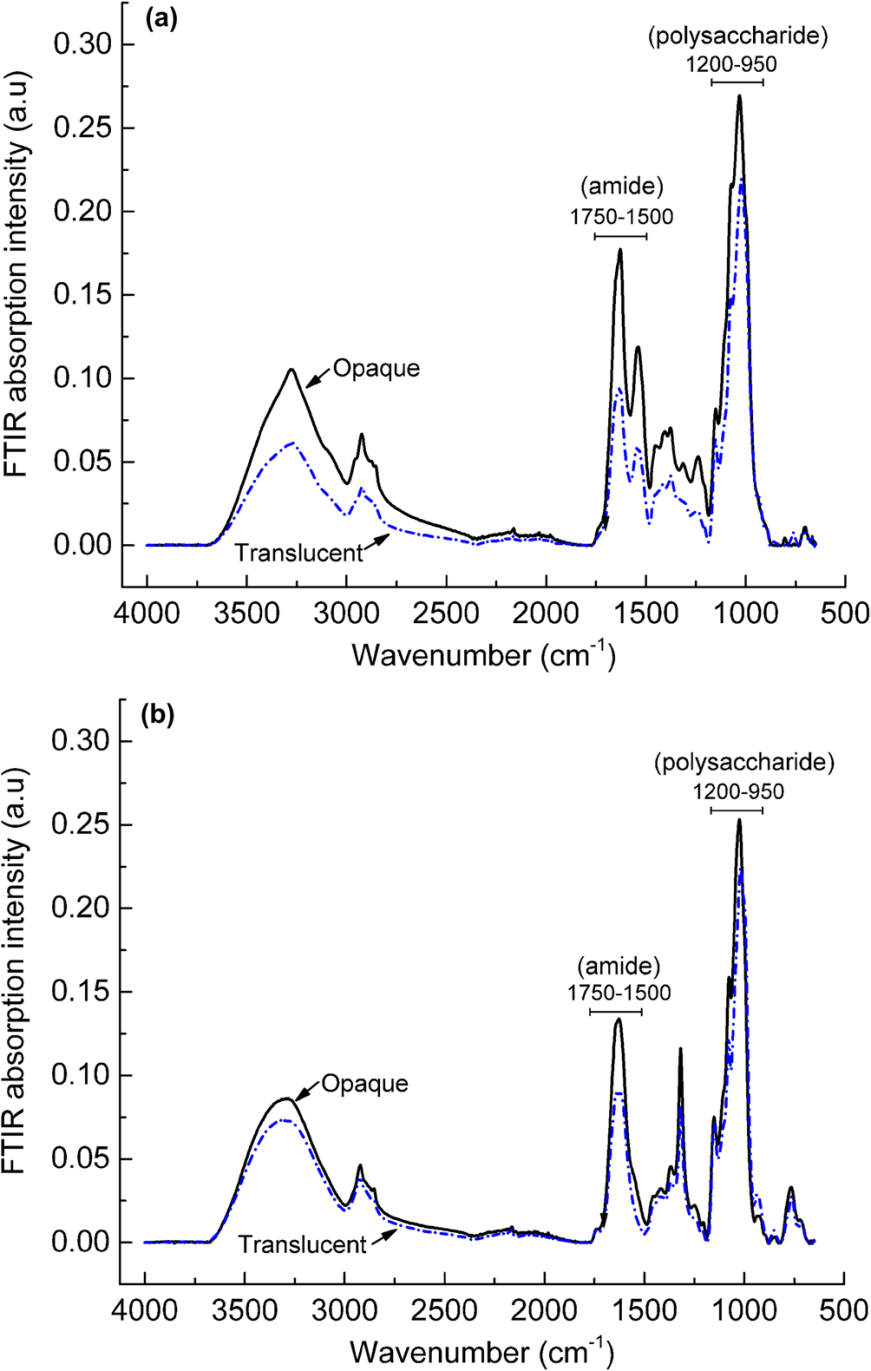
FTIR spectra of the opaque (solid) and translucent (dot-dash) material extracts from pristine mycelia species of *Pleurotus ostreatus* (**a**) and *Trametes versicolor* (**b**).

Despite the relatively low overall mass yield contribution from the translucent sections, exploring the biochemical structural differences between the translucent and opaque hyphae material was critical. Variations in the biochemical structures between the opaque and translucent materials can significantly influence their thermal stability. Only the FTIR spectra of the opaque and translucent material extracts from *Pleurotus ostreatus* and *Trametes versicolor* are presented in Fig. 6 since *Ganoderma australe* did not have translucent material. The relatively lower computed ratios of the integrated area under the protein peak (1750–1500 cm^−1^) and the primary carbohydrate peak (1200–950 cm^−1^) suggest reduced *N*-acetyl-*d*-glucosamine content (i.e., chitin) within the translucent sections. The reduced content of carbon-rich chitin polysaccharides can adversely impact the generation of carbonaceous char leading to relatively lower residual char yields for the translucent material.

**Figure 7:**
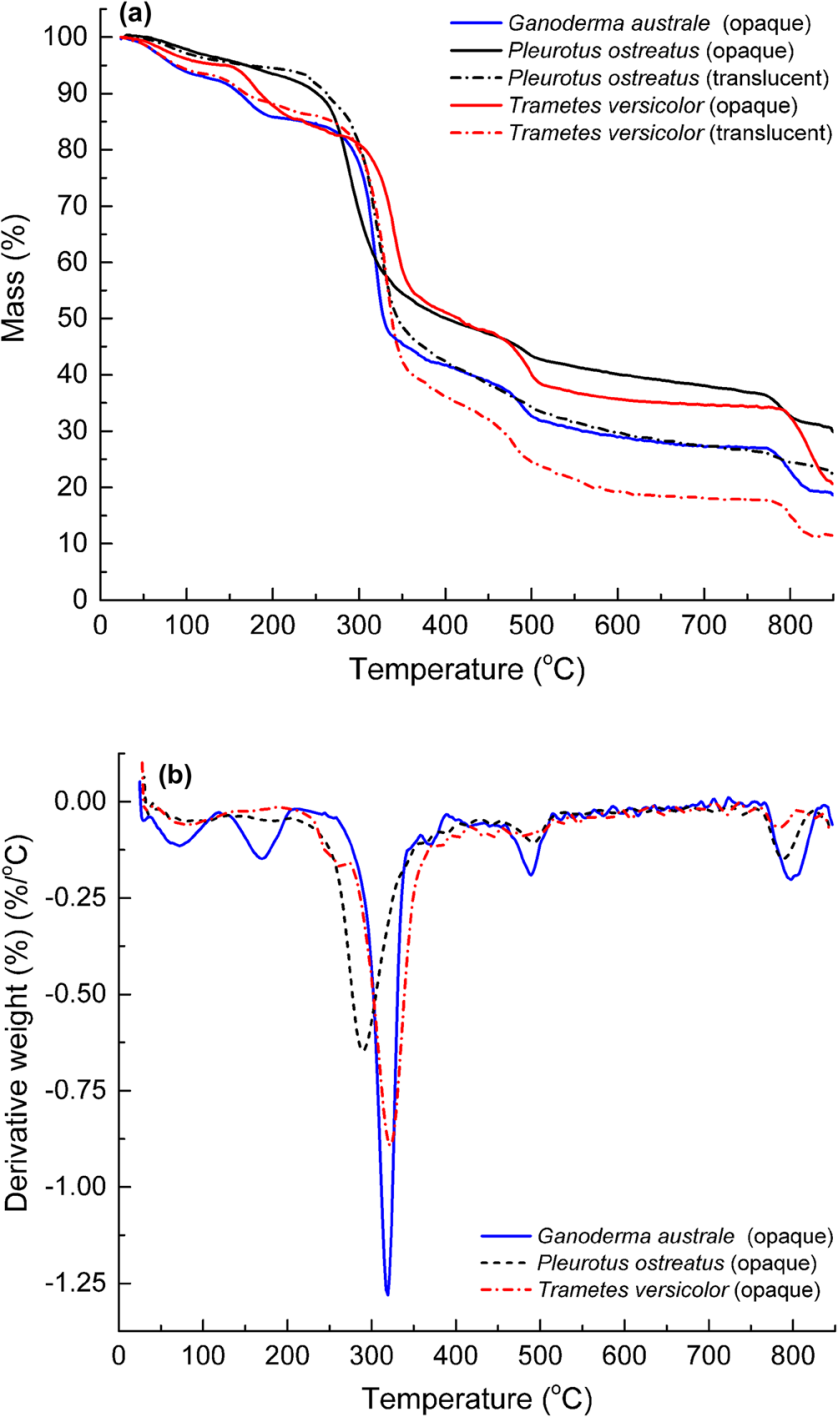
(**a**) Thermogravimetric mass loss and (**b**) mass loss rate profiles of mycelia species *Ganoderma australe*, *Pleurotus ostreatus* and *Trametes versicolor*.

Thermogravimetric analysis was conducted to establish the relationship between fungal species growth rates, biochemical composition, and thermal properties. Thermogravimetric mass loss (TG) and the derivative thermogravimetric mass loss rate (DTG) profiles simultaneously measured for the mycelia species are shown in Fig. 7. In the case of *Pleurotus ostreatus and Trametes versicolor*, test samples were extracted from both the opaque and translucent sections to investigate the influence of material composition on thermal stability. Samples extracted from the opaque and translucent sections of *Pleurotus ostreatus and Trametes versicolor* species had distinct thermal degradation profiles. The thermal stability of the opaque material was superior to that of the translucent section. The higher carbon-rich chitin content in the opaque material, as revealed by the FTIR spectra (Fig. 6), is possibly responsible for the superior thermal stability.

Since the opaque material was present in all fungal species, the discussion on thermal stability focused on this material. The TG and DTG profiles of all three fungal species revealed multi-step mass losses (8–15%) between 25 and 225 °C ahead of the onset of the main thermal degradation stage. Given that all mycelia specimens were conditioned at 60 °C for 3 h immediately before the TGA experiment, the atypical early mass loss could not be attributed to the desorption of physically adsorbed water. Instead, the low-temperature mass loss was ascribed to thermal degradation and volatilisation of the low molecular weight (< 20 kDa) hydrophobins layer^22^. The initial thermal degradation stage (25–225 °C) was followed by the main thermal decomposition stage (225–450 °C), over which all mycelia species registered mass losses between 35 and 45%. The mass loss between 225–450 °C was attributed to the thermal decomposition of the fungal cell wall consisting of chitin, amino acids, carbohydrates including polysaccharides, and glucan. Compared to *Ganoderma australe*, *Pleurotus ostreatus* and *Trametes versicolor* species had higher thermal stability possibly due to their higher chitin content evident from the strong *N*-acetyl-*d*-glucosamine-induced FTIR absorption band at 1750–1500 cm^−1^. Chitin is a rich source of elemental carbon necessary for char formation. Substantial mass losses (6–14%) between 400 and 650 °C were recorded for all three mycelia species and attributed to the formation of a primary carbonaceous char. The primary char was stable until just below 800 °C. Beyond 800 °C, continued thermal exposure caused further mass losses (e.g., 7–14%) generating secondary carbonaceous char. The secondary carbonaceous char is a product of cross-linking in the primary char to form a consolidated 3D network at high temperatures. *Pleurotus ostreatus* had the highest secondary residual char yield at 850 °C (30%) followed by *Trametes versicolor* (22%) with *Ganoderma australe* having the lowest yield (20%) (Fig. 7a). It is noteworthy that the fastest-growing *Ganoderma australe* species was the least thermally stable fungal species. On the other hand, the most thermally stable *Trametes versicolor* species grew the slowest. These observations suggest an inverse proportional relationship between mass yields and thermal stability. Retarded fungi growth rates possibly allow time for the establishment of chitin which in turn promotes char formation. Both the growth rates and thermal properties are critical in the selection of fungal species for high throughput production of bio-based fire retardants.

**Figure 8:**
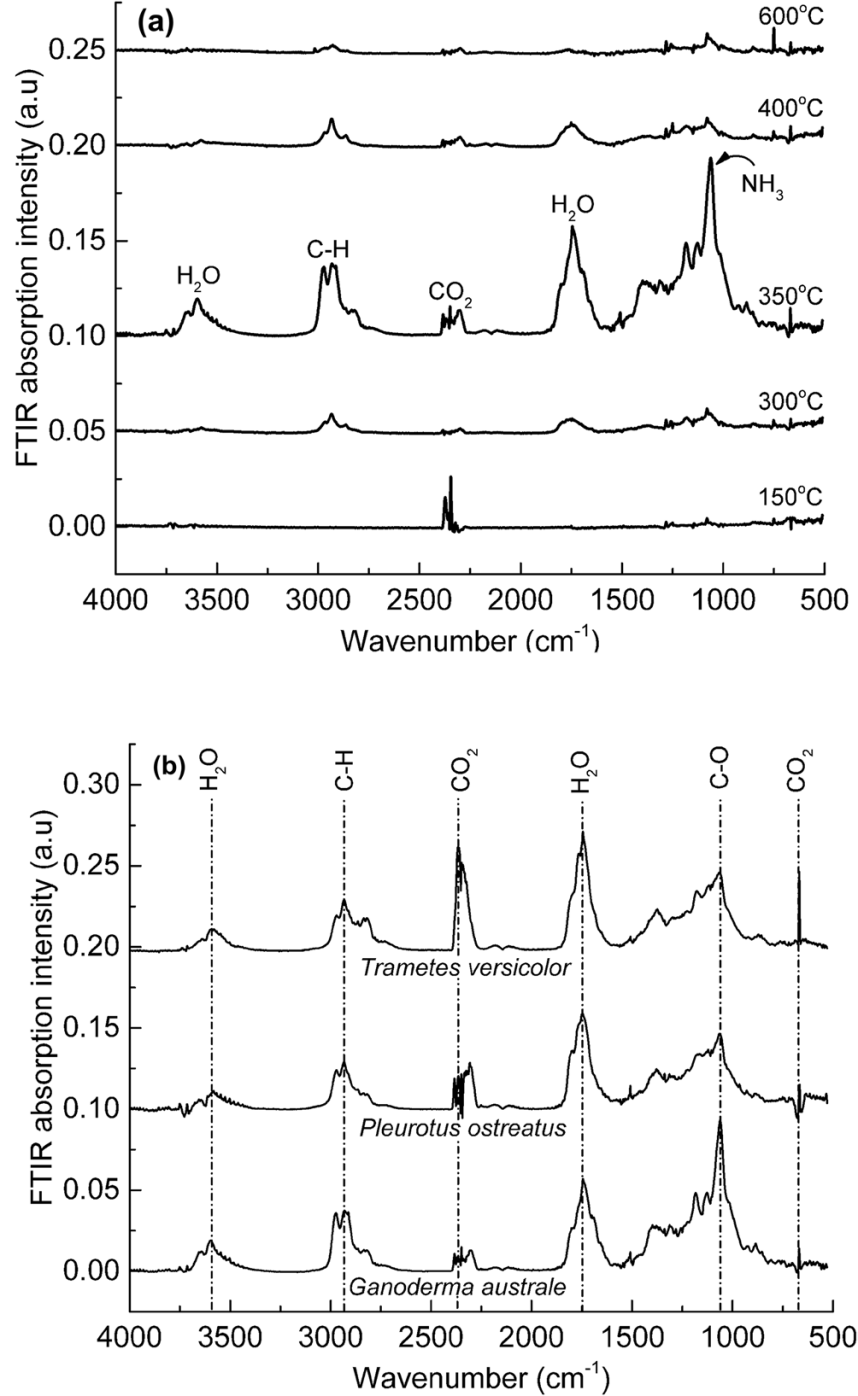
FTIR spectra of (**a**) gases evolved at different thermal decomposition temperatures (150, 300, 350, 400 and 600 °C) for *Ganoderma australe* and (**b**) gases evolved from all three fungal species at 350 °C. FTIR spectra were offset along the Y-axes but not scaled.

While there are some variations in the mass loss-temperature profiles of the mycelia species (Fig. 7a), overall, the mass loss and mass loss rates suggest comparable thermal degradation mechanisms and thermal stability. To ascertain the mechanisms governing the thermal degradation, a TGA-interfaced Fourier-transform infrared spectrometer was used to analyse the evolved gases in real-time. Since the evolved gas FTIR spectra between the three fungal species were similar, only the TGA-FTIR data collected for *Ganoderma australe* is discussed. The FTIR spectra of the gases evolved from *Ganoderma australe* at selected decomposition temperatures of 150, 300, 350, 400 and 600 °C are shown in Fig. 8a. FTIR spectrum collected at 150 °C revealed the release of CO_2_ which may be attributed to thermal oxidation of low molecular weight hydrophobins. The FTIR spectrum of gases evolved at the onset of the major decomposition stage, at approximately 300 °C, revealed the presence of H_2_O, possibly low molecular weight hydrocarbons such as CH_4_, minute amounts of CO_2_, and NH_3_. The intensities of the IR bands corresponding to CO_2_, H_2_O, NH_3_ and the low molecular weight hydrocarbons peaked at the decomposition temperature of 350 °C (e.g., the tail-end of the significant thermal decomposition stage). Above 350 °C, the FTIR intensities decreased with increasing temperatures as evident from a near-flat spectrum for gases evolved at 600 °C. The FTIR spectra of gases evolved at the thermal decomposition temperature of 350 °C for all three species are presented in Fig. 8B. There were variations in the intensity but no difference in the evolved gas composition between the three fungal species. The diluent gases (i.e., CO_2_, H_2_O and NH_3_) identified in the FTIR spectra of the three mycelia species are critical in dampening the flaming combustion reactions. They can promote fire suppression when mycelium is integrated with highly flammable materials such as olefinic polymers.

The residual char collected at 600 °C was characterised using SEM and FTIR to elucidate the thermal degradation mechanisms. The post-thermal exposure SEM images for *Ganoderma australe, Pleurotus ostreatus and Trametes versicolor* shown in Fig. 4 revealed discernible microstructure variations to the pristine specimens. *Ganoderma australe* retained its fused and interconnected hyphae microstructure despite the reduction in the cross-sectional area of the hyphae strands (Fig. 4d). In the case of *Pleurotus ostreatus* (Fig. 4e) and *Trametes versicolor* (Fig. 4f), the fungal species retained the 3D hyphae network, even though the hyphae cross-section was significantly reduced due to thermal-induced material loss. The highly thermally stable chitin in the cell walls of mycelium continued to support the 3D fibrous network despite the reduction in hyphae filament diameter^24^. These results agree with the findings reported by Jones et al.^24^ for wheat-fed *Trametes versicolor* subjected to similar thermal degradation conditions. Despite some variations in the post-thermal exposure microstructure, the residual char yields across the three distinct fungal species were similar. This suggests that microstructural characteristics of the pristine mycelia have limited impact on their thermal decomposition mechanisms. This is supported by the findings reported by Jones et al.^24^ in which they demonstrated that the growth time-dependent microstructure variations in the wheat-fed *Trametes versicolor* did not alter the fire reaction properties evaluated via the cone calorimeter.

**Figure 9:**
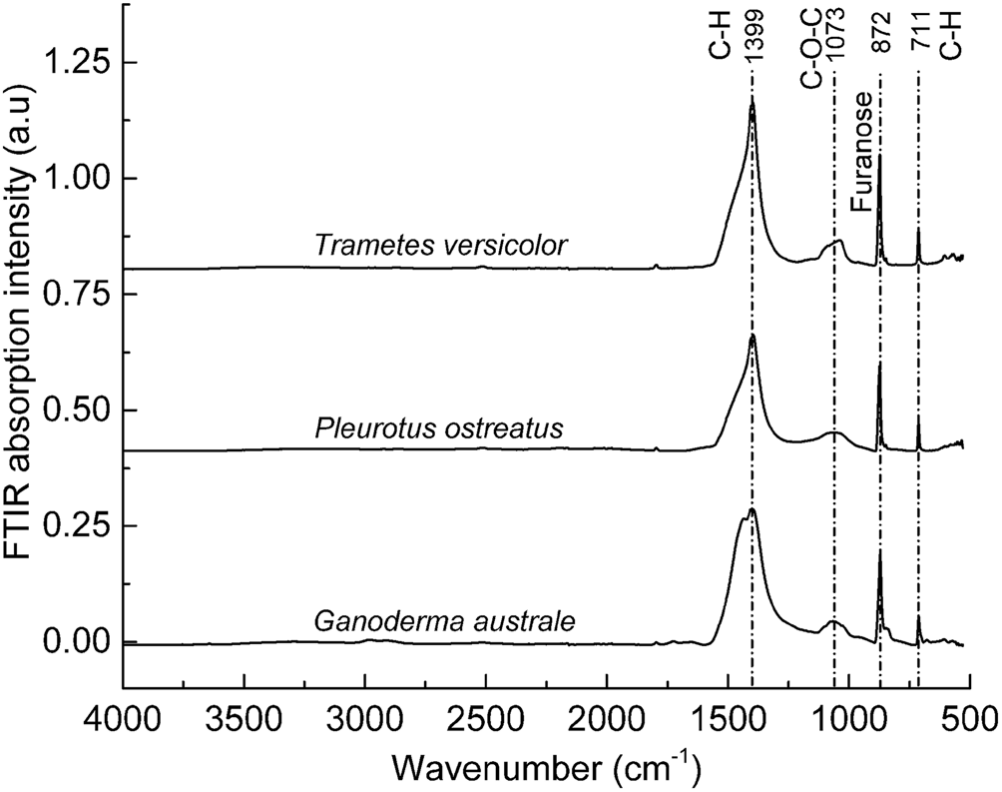
FTIR spectra of residual char collected from mycelia species *Ganoderma australe*, *Pleurotus ostreatus* and *Trametes versicolor* at 600 °C. FTIR spectra are offset along the Y-axes but not scaled.

FTIR spectra of the char residue recovered from the opaque mycelia specimens at 600 °C (i.e., primary char) are shown in Fig. 9. All mycelia species exhibited the following absorption bands; strong and sharp peaks centred at ~ 1400 cm^−1^ (C–H bending)^43^ and ~ 870 cm^−1^ (C–H bending furanose ring)^44^, a weak broad peak centred at ~ 1030 cm^−1^ (C–O–C stretching)^33^, 950–750 cm^−1^ (glycoside bonds in glucan)^33,43^ and a weak but sharp peak at ~ 710 cm^−1^ (C–H bending)^43^. The furanose ring may be attributed to ring-chain tautomerism of pyranose due to high-temperature exposure. There were no distinct differences in the room temperature measured FTIR spectra of recovered char from the three species; further reinforcing the finding that microstructural variations have very little influence on the thermal degradation pathways and decomposition products including the residual char.

**Figure 10:**
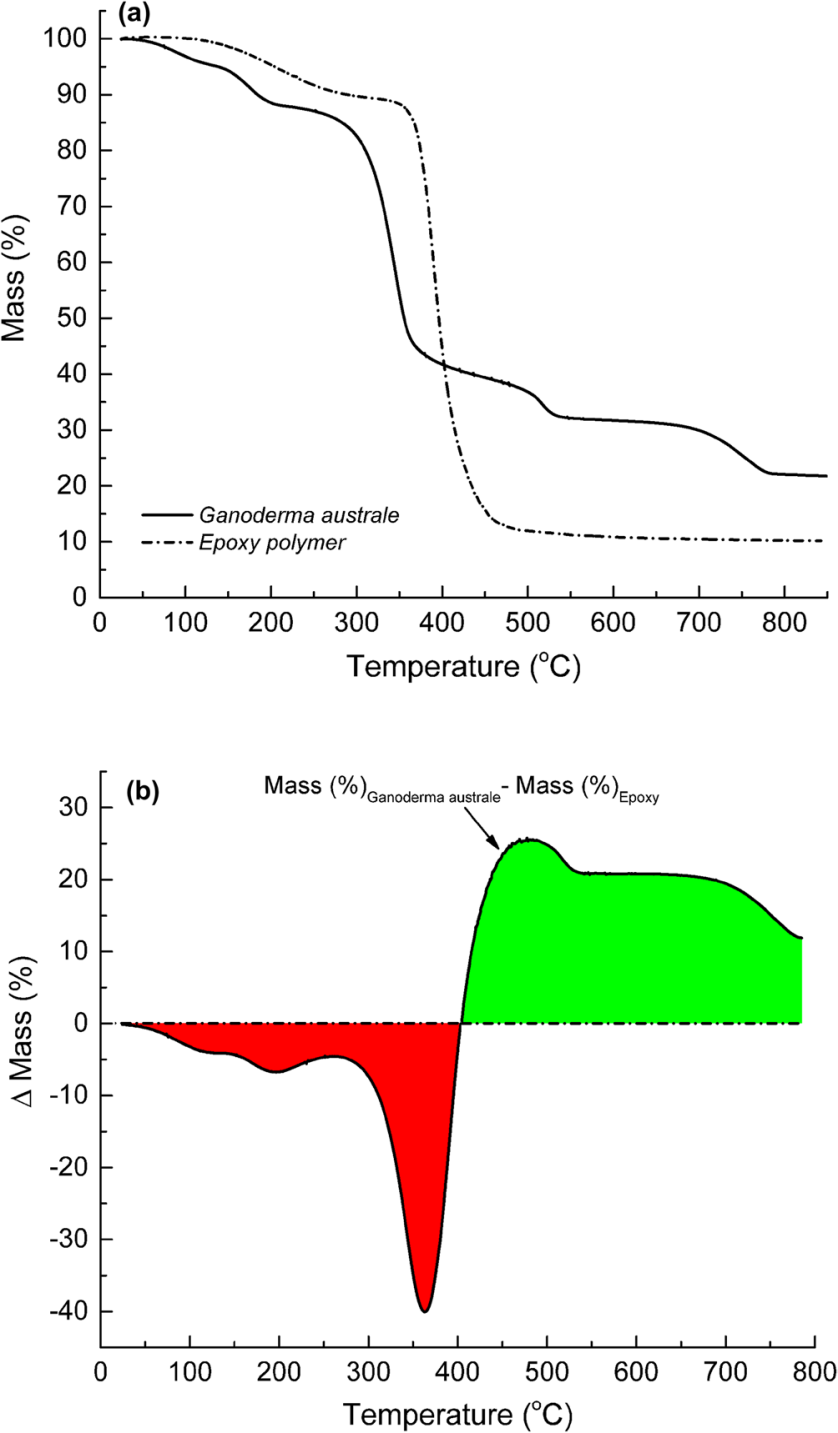
(**a**) Thermogravimetric mass loss for *Ganoderma australe* and epoxy and (**b**) mass difference (*Garnoderma australe* minus epoxy) profiles plotted against temperature (loss in red and gain in green).

Before mycelia can be integrated into engineering composites as a fire retardant, it is critical to understand how this novel biomaterial compares to common composite matrices such as epoxy resins. In this work, the most productive mycelia species, *Ganoderma australe*, was benchmarked against an epoxy polymer. The mass loss and mass difference profiles of *Ganoderma australe* and epoxy polymer are plotted against temperature in Fig. 10. The epoxy polymer followed a two-step thermal degradation pathway; a 10% mass loss between 100 and 250 °C followed by approximately 80% mass loss between 300 and 500 °C (Fig. 10a). The first thermal mass loss stage was attributed to desorption of physically adsorbed moisture and under cross-linked polymer moieties. The more prominent second step was attributed to the thermal decomposition of the epoxy polymer chains. At 250 °C, *Ganoderma australe* had lost 4–5% more material compared to the epoxy polymer. At temperatures below 405 °C, the thermal stability of the epoxy polymer was superior to that of *Ganoderma australe*. The same was true for *Pleurotus ostreatus* and *Trametes versicolor*, both of which had similar thermal degradation profiles to *Ganoderma australe*. At temperatures above 405 °C, *Ganoderma australe*, and by extension *Pleurotus ostreatus* and *Trametes versicolor* was thermally more stable than the epoxy polymer. The fungal species generated significantly higher primary residual char yields (> 30%) at 600 °C compared to approximately 10% for the epoxy polymer.

The difference in the thermal stability between *Ganoderma australe* and the epoxy polymer is more apparent in the mass difference data (*mass*_*Ganoderma australe*_ − *mass*_*epoxy*_) which is plotted against the temperature in Fig. 10b. For temperatures below 405 °C, the epoxy polymer was thermally more stable than *Ganoderma australe*, as evident from the negative mass differences. Above 405 °C, *Ganoderma australe* was superior to the epoxy polymer as revealed by the positive computed mass differences. The integrated area under the mass difference-temperature profile for temperatures between 405 and 785 °C (green shade) was more than twice greater than the corresponding value calculated for temperatures between 25 and 405 °C (red shade). Notably, the integrated temperature range to the left and the right of the cross-over (405 °C) was the same, suggesting an overall superior thermal stability for *Ganoderma australe* compared to the epoxy polymer. The enhanced thermal stability of the mycelia fungal species at relatively high temperatures, possibly due to the presence of chitin and char promoting calcium derivates, opens an avenue to the design of bio-derived thermal protective surface materials for polymer composites threatened by fire. When exposed to high radiant heat fluxes, the temperature at the exposed surface of a polymer composite can rise beyond 400 °C in a matter of seconds. Thermal degradation of the mycelium protective layer will begin at temperatures below 100 °C. As the mycelia surface protective film decomposes, it will generate consolidated surface char serving to thermally insulate the underlying virgin but combustible polymer composite.

**Figure 11:**
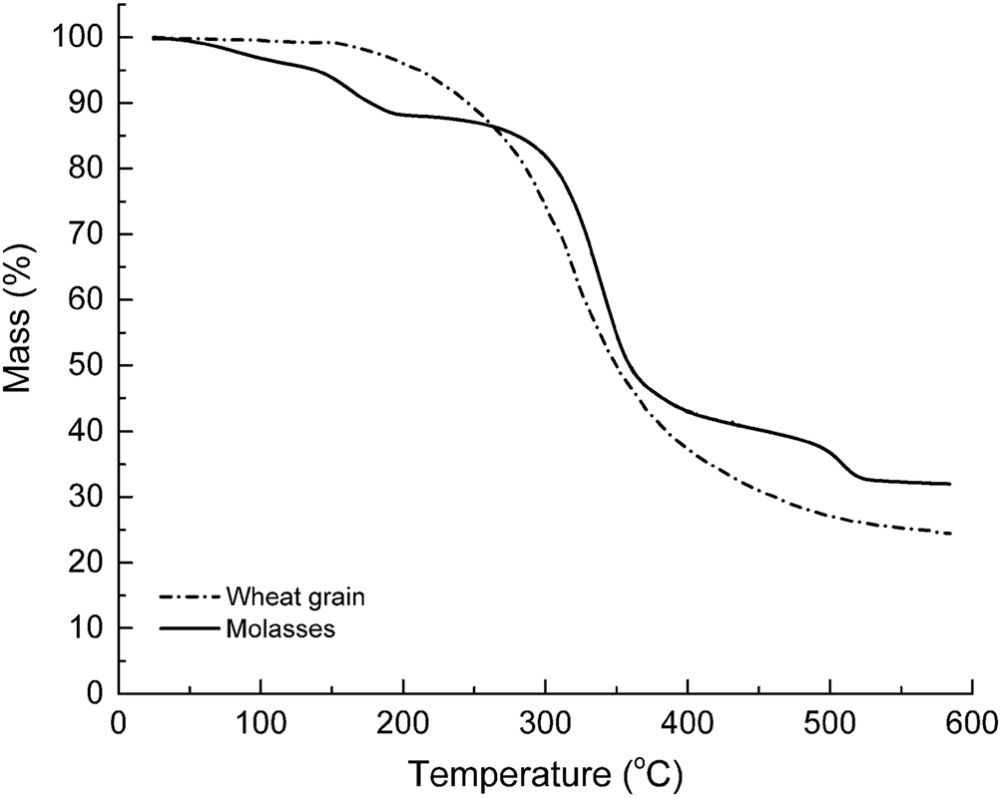
Mass loss-temperature profiles of *Trametes versicolor* fed molasses [this work] and wheat grain^24^.

There are very few studies that have investigated the influence of molasses or similar liquid-phase feed on the growth rate, yields as well as the thermal stability of resultant mycelia. When solid feed material is used to grow mycelium, the partially digested residual feed material cannot be separated from the final biocomposite. The residual solid feed material can adversely affect the mechanical and fire properties of the mycelia biocomposite. It was, therefore, important to investigate the variations in thermal stability between mycelia species independently grown in solid wheat grain and molasses. The thermal properties of the molasses-fed *Trametes versicolor* were compared to those of the same species grown in solid wheat grain by Jones et al.^24^ as shown in Fig. 11. At temperatures below 250 °C, the molasses-fed fungi was inferior to the wheat grown specimen. However, at temperatures greater than 250 °C, the molasses fed fungi specimen was more thermally stable. Like wheat grain, molasses can support the growth of mycelium fungi producing biomaterials of comparable if not superior thermal stability particularly in the high-temperature regime (> 250 °C). Further, while wheat grain particles become inextricably integrated with mycelium hyphae, excess molasses can be washed off. Jones et al.^24^ demonstrated that the thermal stability of wheat grain is inferior to that of *Trametes versicolor*. Therefore, the presence of the less thermally stable wheat grain particles^24^ has the potential to reduce the fire protection efficacy as well as the mechanical properties of the resultant mycelium composite. Further, this study confirmed that the recovered molasses solution could support the growth of fresh inoculum albeit at slightly diminished growth rates. In contrast, it is not possible to recover residual solid feed material to support future mycelia cultivation.

For industrial-scale applications, large mycelium films grown in shallow trays filled with molasses can be integrated as thermal protective surface layers on fire-threatened flammable materials and composites. Mycelia films can act as the sacrificial material, thermally decomposing to produce secondary carbonaceous char on the fire-exposed surface. The generated surface char can slow down heat transfer into the underlying flammable substrate while preventing combustible volatiles from escaping into the flaming-combustion zone. Alternatively, mycelium biomass can be pulverised into micro-particles which are then mixed into the polymer matrix. While the integration of micro-sized FRs into polymers is well established, it is noteworthy; that the addition of powdered or micro-fibres of mycelium into the polymer can increase the viscosity resulting in material processing issues. Nevertheless, we have demonstrated in this paper that molasses is a viable feed for cultivating thin mycelium films with promising thermal stability and possibly fire-retardant characteristics. Further, research should focus on developing methodologies for integrating mycelium films into industrial-scale manufacturing processes for fire-retarded products.

## Conclusion

An environmentally benign method for upcycling low-value sugar processing by-product (molasses) into fire retardant mycelia is reported. This research demonstrated that liquid molasses supports the growth of non-pathogenic *Basidiomycota* phylum mycelia species (*Ganoderma australe, Pleurotus ostreatus and Trametes versicolor*). *Ganoderma australe* grew the fastest producing an opaque mycelium film and had the highest mass yield. *Pleurotus ostreatus and Trametes versicolor* had lower mass yields and produced mycelium films with opaque and translucent sections. Despite the differences in the morphology and microstructural characteristics, the biochemical compositions of the investigated fungal species were comparable. This study established the relationship between mycelia growth rates, microstructures, biochemical compositions and thermal properties.

The fungal species with highest growth rate, *Ganoderma australe*, was the least thermally stable of all three species. The varied growth rates and different microstructural features influenced the thermal degradation (mass loss versus temperature) profiles of the three species to a limited extent. Similar FTIR spectra of the evolved gases and residual primary char suggested comparable thermal degradation mechanisms for the three fungal species. The thermal stability of mycelia is possibly controlled by the chitin content whose yield maybe dictated by the fungal growth rate. The molasses-fed mycelia generated more surface char than the petroleum-based epoxy polymer, suggesting potential use of the fungi-derived biocomposite as a thermal protective film. The thermal stability of the *Trametes versicolor* species grown in molasses was superior to the wheat-fed counterpart. This study demonstrated the feasibility of producing fungal biomaterials with superior char forming capabilities compared to commercial polymers like epoxy. The findings reported in this paper will pave the way for the development of new sustainable and fire-retardant biomaterials.
